# Integrative analysis of congenital muscular torticollis: from gene expression to clinical significance

**DOI:** 10.1186/1755-8794-6-S2-S10

**Published:** 2013-05-07

**Authors:** Shin-Young Yim, Dukyong Yoon, Myong Chul Park, Il Jae Lee, Jang-Hee Kim, Myung Ae Lee, Kyu-Sung Kwack, Jan-Dee Lee, Jeong-Hun Lee, Euy-Young Soh, Young-In Na, Rae Woong Park, KiYoung Lee, Jae-Bum Jun

**Affiliations:** 1 The Center for Torticollis, Department of Physical Medicine and Rehabilitation, Ajou University School of Medicine, Suwon, Republic of Korea; 2 Department of Biomedical Informatics, Ajou University School of Medicine, Suwon, Republic of Korea; 3 Department of Plastic and Reconstructive Surgery, Ajou University School of Medicine, Suwon, Republic of Korea; 4 Department of Pathology, Ajou University School of Medicine, Suwon, Republic of Korea; 5 Brain Disease Research Center, Ajou University School of Medicine, Suwon, Republic of Korea; 6 Department of Radiology, Ajou University School of Medicine, Suwon, Republic of Korea; 7 Department of Surgery, Yonsei University College of Medicine, Seoul, Republic of Korea; 8 Department of Surgery, Ajou University School of Medicine, Suwon, Republic of Korea; 9 Department of Rheumatology, The Hospital for Rheumatic Diseases, Hanyang University College of Medicine, Seoul, Republic of Korea

## Abstract

**Background:**

Congenital muscular torticollis (CMT) is characterized by thickening and/or tightness of the unilateral sternocleidomastoid muscle (SCM), ending up with torticollis. Our aim was to identify differentially expressed genes (DEGs) and novel protein interaction network modules of CMT, and to discover the relationship between gene expressions and clinical severity of CMT.

**Results:**

Twenty-eight sternocleidomastoid muscles (SCMs) from 23 subjects with CMT and 5 SCMs without CMT were allocated for microarray, MRI, or imunohistochemical studies. We first identified 269 genes as the DEGs in CMT. Gene ontology enrichment analysis revealed that the main function of the DEGs is for extracellular region part during developmental processes. Five CMT-related protein network modules were identified, which showed that the important pathway is fibrosis related with collagen and elastin fibrillogenesis with an evidence of DNA repair mechanism. Interestingly, the expression levels of the 8 DEGs called CMT signature genes whose mRNA expression was double-confirmed by quantitative real time PCR showed good correlation with the severity of CMT which was measured with the pre-operational MRI images (R^2 ^ranging from 0.82 to 0.21). Moreover, the protein expressions of ELN, ASPN and CHD3 which were identified from the CMT-related protein network modules demonstrated the differential expression between the CMT and normal SCM.

**Conclusions:**

We here provided an integrative analysis of CMT from gene expression to clinical significance, which showed good correlation with clinical severity of CMT. Furthermore, the CMT-related protein network modules were identified, which provided more in-depth understanding of pathophysiology of CMT.

## Background

Congenital muscular torticollis (CMT) is one of the most common musculoskeletal problems in children. The incidence of CMT has been reported to be as high as 3.92% in neonates [1,2]. From the clinical point of view, CMT is characterized by a thickening and/or tightness of the unilateral sternocleidomastoid muscle (SCM), and the end result is limited neck motion [2,3]. Currently we believe that CMT may be caused by different mechanisms at different stages of either the prenatal or perinatal period. CMT is known not to occur in the postnatal period, indicating that some characteristics of the fetal developing muscles are critical for the development of CMT. However, our understanding of the CMT characteristics is limited. Moreover, the pathogenesis and pathophysiology of CMT still remains elusive while fetal mal-positioning in the uterus, birth trauma and ischemia of the SCM during either the prenatal or perinatal period have been widely proposed as the causes of CMT [4-6].

Currently, the main histopathologic finding of CMT is known to be interstitial fibrosis [7,8]. Fibrosis leads to a reduction of the mobile proton density, and so this would show hypointensity on both the T1- and T2-weighted magnetic resonance images (MRI). If the fibrosis is more localized, then localized low signal intensity is observed. If the fibrosis is diffuse, then the entire SCM muscle shows diffuse hypointensity. About 90% of the cases of CMT are known to be cured with only stretching exercises without causing any musculoskeletal complications since they have small amount of fibrosis [9]. However, about 10% of the cases of CMT have a large amount of dense connective tissue within the SCM, and this shows more hypointensity on both the T1- and T2-weighted MRIs. This is the most severely involved subgroup of CMT and these cases hardly respond to stretching exercises. Therefore, surgical release is needed for this subgroup of CMT in order to minimize the secondary musculoskeletal complications of CMT.

Meanwhile, microarray has emerged as a fundamental tool for studying gene expressions and genome-wide microarray expression analysis is a useful tool for getting a comprehensive picture of the gene expression at the molecular level, even without having prior knowledge of the major genes or susceptibility loci for a certain disease [10]. To the best of our knowledge, however, there has been no previous study that has investigated the gene expression signature of CMT.

Here we tried to discover the novel pathways related with the pathophysiology of CMT through identifying a gene and protein expression signature of the subjects with CMT who needed surgical release. The objectives of this study were 1) to identify a gene expression signature that was differentially expressed in the tissue with CMT compared to the tissue without CMT, 2) to discover the novel protein interaction network modules related with the pathophysiology of CMT, 3) to identify correlation between the gene expression profiling and the clinical severity of CMT, 4) to demonstrate expression of the proteins encoded by the differentially expressed genes (DEGs) in CMT tissue; and 5) to provide an integrative analysis of gene expression profiling with clinical severity of CMT and the expression of proteins encoded by DEG.

## Methods

### Acquisition of the tissue with CMT (T-CMT) and the tissue without CMT (T-control)

This study was approved by the Institutional Research Board of Ajou University Medical Center, Suwon, South Korea. Twenty-eight subjects (23 subjects with CMT and 5 subjects without CMT) were finally enrolled in this study. For the microarray or quantitative real-time PCR (QRT-PCR) and MRI analyses, 26 subjects with CMT originally participated in this study. Myectomy was done at the lower end of the SCM about 1 centimeter above the clavicle. All the 26 subjects who agreed to participate in this study understood that the removed muscle blocks from the surgery would be used for the analyses. Each muscle block from 26 subjects was divided into the T-CMT and the T-control by the third author's visual assessment of their gross appearance. While the reddish part with a normal muscle appearance was considered as T-control, the whitish cord-like part was considered as T-CMT. Since the muscle blocks of 4 subjects had only T-CMT, those subjects were not enrolled. Another 4 subjects showed poor quality of their RNA and they were also excluded. Therefore, 18 subjects were finally enrolled in these analyses. For the imunohistochemical (IHC) analysis, additional 5 subjects with CMT were enrolled. Muscle block were obtained as the same procedure described above. Independent normal SCM muscles for the IHC were obtained from the Ajou Human Bio-Resource Bank, which provided the normal SCM muscle of 5 subjects who underwent radical neck dissection for the head and neck tumor.

### RNA isolation from both the T-CMT and the T-control

The muscle tissue was stored in RNA*later *RNA Stabilization Reagent (Applied Biosystems/Ambion, Austin, TX, USA) immediately after surgery to preserve the RNA. The total RNA was isolated from the tissue using TRIzol reagent (Invitrogen, Carlsbad, CA, USA) according to the manufacturer's instructions.

### Genome-wide mRNA expression profiling using microarray

Seven paired microarray experiments (a total of 14) were done using an Affymetrix GeneChip Human Gene 1.0 ST Array (Affymetrix, Santa Clara, CA, USA) which offers whole-transcript expression profile. 300ng of total RNA extracted from each sample was used as input into the Affymetrix procedure as recommended by the manufacture's protocol (http://www.affymetrix.com). Robust Multiarray Averaging (RMA) method was used for microarray normalization and summarization. When multiple probes per gene were available, we averaged the values of corresponding probes. We applied a quantile normalization method across samples.

### Identification of the DEGs of CMT

The fold change of the expression level was calculated as follows:

$$\begin{array}{c} Fold\;change=\;T-CMT^{*}/T-control^{\text{†}}\;\;if\;T-CMT^{*}>T-control^{†}\\ Fold\;change\;=-\left(T-control^{†}/T-CMT^{*}\right)\;\;if\;T-CMT^{*}\leq T-control^{†}, \end{array}$$

where T-CMT^* ^means the expression level of a gene of the T-CMT, and T-control^† ^means the expression level of the same gene of the T-control. DEGs were identified when genes met the following two conditions at once: 1) genes showing a significant difference of expression between the T-CMT and the T-control (*p *< 0.05; using a Student *t *test), and 2) genes showing more than |2| fold change between the T-CMT and the T-control in more than half subjects (here, >3).

### Examination of the discriminant power of the DEGs between the T-CMT and the T-control

Principal component analysis (PCA) was done using MATLAB R2007a (MathWorks Inc., Natick, MA, USA). The clustering methods such as a k-means clustering method and a hierarchical clustering method were done using the TM4 software [10].

### Gene ontology enrichment analysis

We used functional annotation tools called DAVID (the Database for Annotation, Visualization and Integrated Discovery) [11], and GOEAST (the Gene Ontology Enrichment Analysis Software Toolkit) [12] to find enriched gene ontology terms in identified DEGs.

### Identification of the CMT-related protein network modules

We set up the protein-protein interactions (PPIs) and protein-DNA interactions (PDIs) for Homo sapiens. For the PPIs, we used the data of Lee et al. [13] and the results of several recent genome-wide studies [14-17]. The PPIs consist of 80,970 interactions among 10,819 human proteins. For the PDIs, we extracted 1,539 interactions using the TRANSFAC database [18]. To discover the CMT-related PPIs or PDIs, the prepared PPIs, PDIs and the list of the identified DEGs from this research were imported into Cytoscape (http://www.Cytoscape.org) with their fold change [19]. MCODE was used to find the CMT-related protein network modules [20]. Network score was calculated based on complexity and density of each sub-graph. A module with more than 1 MCODE score was considered significant. Post filtering was performed to remove low-quality modules. In the filtering process, the part of each module shown consistent expression and high connectivity were selected as a final module through manual review. Finally, modules which include at least one protein encoded by DEG and other proteins their association with CMT was previously known were selected as CMT-related protein network modules. For selected five CMT-related modules, network ontology analysis (NOA) published by Wang et al. which perform gene ontology analysis on network module was conducted to define function of the five CMT-related modules [21]. A GO term of which p-value was less than 0.1 was considered significant.

### Quantitative real-time PCR of DEGs

Genome-wide mRNA expression profiling using microarray was validated by QRT-PCR. We selected 8 among 269 DEGs for the validation. 7 were DEGs which show top difference expression in fold change and the *t*-test (*p *value) in the microarray study. The other one is S100A4, which was a component of one of CMT-related protein network modules and showed key role as the first split point in decision tree model discriminating T-CMT and T-control (Figure S1 in Additional file 1). Eight DEGs were as follows: *thrombospondin 4 (THBS4), fibromodulin (FMOD), collagen*, *type XIV, alpha 1 (COL14A1)*, *cathepsin K (CTSK)*, *epidermal growth factor (EGF)-like repeats and discoidin I-like domains 3 (EDIL3)*, *lysyl oxidase (LOX), secreted frizzled-related protein 4 (SFRP4)*, and *S100 calcium binding protein A4 (S100A4)*. QRT-PCR was done for 11 paired (T-CMT and T-control) muscle tissues (a total of 22 specimens) from 11 independent subjects. *Glyceraldehyde-3-phosphate dehydrogenase (GAPDH) *was used as an internal control. R^2 ^of the linear regression analysis was used for evaluating the degree of correlation between the microarray and the QRT-PCR expression levels. See the Table S1 in Additional file 1 for the primers used here.

### Correlation between the expression levels of QRT-PCR and the color intensity of lesions on MRI

Eleven subjects with CMT were recruited in both QRT-PCR study and the pre-operational MRI study. The fold changes from the QRT-PCR described above were used as the gene expression level. The difference of grey color intensity between the SCM with CMT (SCM-CMT) and the contralateral SCM (SCM-control) on the pre-operational neck MRI was used as an indicator of the radiological severity. In terms of pathology, CMT is interstitial fibrosis with/without aberrant tendon-like dense connective tissue. Fibrosis will lead to a reduction of the mobile proton (hydrogen ion) density, and so this will show as a darker grey color with a lower scale on both the T1- and T2-weighted MR images. Thus, darker grey color with a lower scale means much fibrosis within the SCM-CMT. We measured the mean intensity of the SCM-CMT on the axial T1 weighted, pre-operational MRI image that showed lowest signal intensity using the region of interest (ROI) method. The mean intensity of the SCM-control was measured on the same axial T1 weighted image using the same ROI. The mean intensity of each SCM was divided by that of its corresponding SCM-control for normalization. Therefore, the difference between the SCM-CMT and the SCM-control was calculated as follows for the independent 11 subjects used in the QRT-PCR analysis:

*Difference of grey color intensity = (the mean intensity of the SCM-control - the mean intensity of the SCM-CMT)/the mean intensity of the SCM-control*.

Next, we compared the QRT-PCR expression level of the identified 8 DEGs with the difference of grey color intensity of the 11 subjects. R^2 ^of the linear regression analysis was used for evaluating their degree of correlation.

### Immunohistochemical examination

For immunohistochemistry, 4-µm thick sections of formalin-fixed, paraffin-embedded tissue blocks were cut from the SCM muscles with CMT and the normal SCM. Sections were deparaffinized in xylene, rehydrated in graded alcohols, followed by antigen retrieval. The endogenous peroxidase activity was blocked by Hydrogen Peroxide Block (Thermo Fisher Scientific, Fremont, CA). Sections were then placed in an automated IHC stainer (Lab Vision Autostainer LV-1; Thermo Fisher Scientific, Fremont, CA) for immunohistochemistry and incubated at 4℃ with primary antibodies against elastin, asporin, CHD3, tenascin, THBS4, EDIL3 (Table S2 in Additional file 1). EDIL3 and ASPN were selected because two were top 2 over-expressed DEGs. The remaining 4 proteins were selected from the CMT-related network modules (ELN, CHD3, TNC, and THBS4). The primary antibodies were detected using the UltraVision LP Detection System (Thermo Fisher Scientific, Fremont, CA). The reaction products were developed with the Vector NovaRED^® ^substrate kit for peroxidase (Vector Laboratories, Burlingame, CA) for 5 minutes, and hematoxylin counterstaining was then applied. The IHC staining was assessed in the CMT and normal SCM. The intensity of IHC staining was scored semi-qauntitatively as follows (0-3): 0, no immune-expression; 1, weak immune-expression; 2, moderate immune-expression; 3, marked immune-expression. The Mann-Whitney test was used to test significance of the intensity of IHC staining between the CMT and normal SCM. *P *values less than 0.05 were considered statistically.

## Results

### The characteristics of the subjects

**Figure 1:**
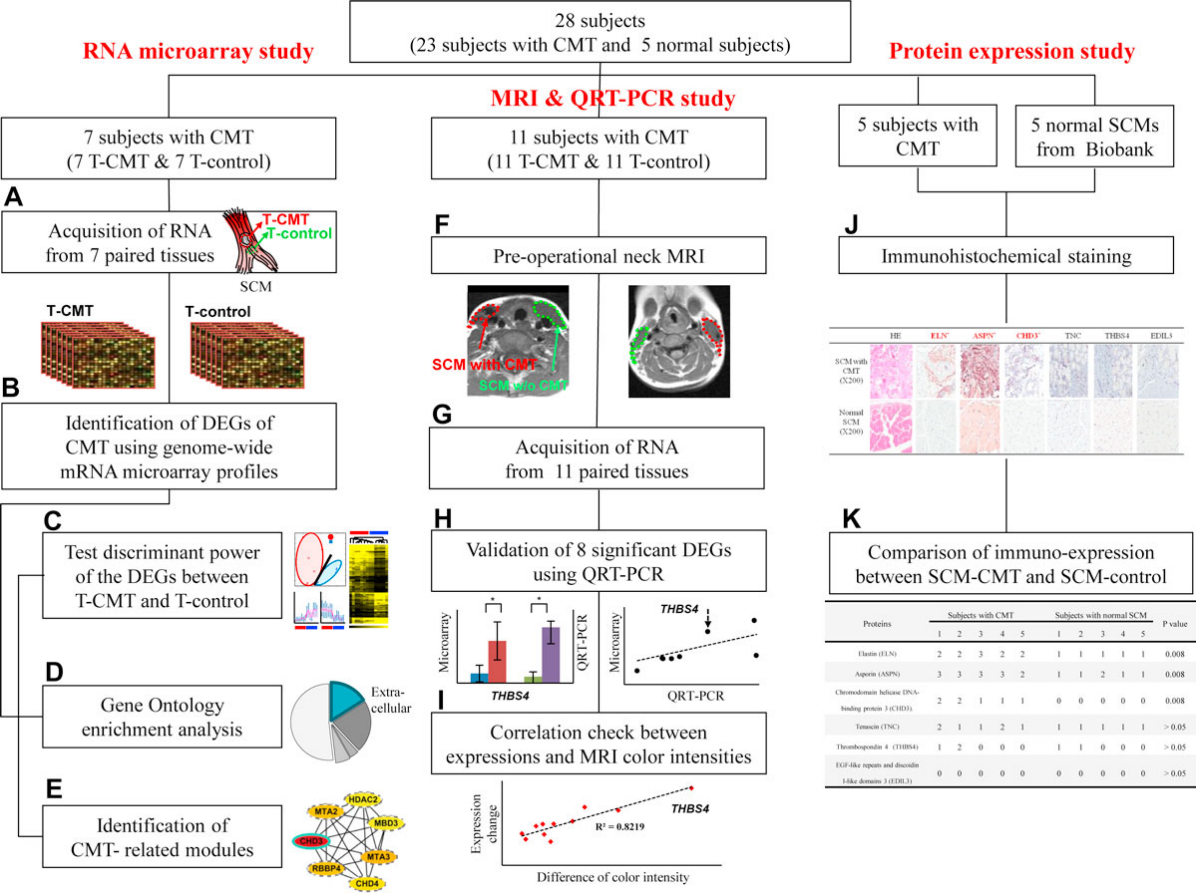
**The overview of the method**. Twenty eight subjects were allocated in the microarray study, the MRI and quantitative real-time PCR (QRT-PCR) study, and immunohistochemical study, respectively. In the microarray study, differentially expressed genes (DEGs) of T-CMT were identified (A-B). The discriminant power of the DEGs (C) was examined and the gene ontology enrichment analysis (D) was done. The CMT-related network modules (E) were identified. In the MRI and QRT-PCR study, the pre-operational neck MRI was taken and analyzed (F-G). The expression level of 8 selected DEGs in T-CMT was measured through the QRT-PCR assay (H). The correlation between the expression level of the DEGs and pre-operational MRI findings was examined (I). The expression of the proteins encoded by the DEGs of CMT was examined using immunohistochemical examination for 5 subjects with CMT compared to the normal SCM muscles from the Bio-resource Bank (J-K).

Twenty-eight subjects were finally enrolled in the RNA microarray study (*n *= 7), MRI and QRT-PCR study (*n *= 11) or IHC studies (*n *= 5 for CMT and *n *= 5 for normal SCM) (Figure 1). The demographic characteristics of the 23 subjects with CMT are presented in Table S3 in Additional file 1. Except for one subject who was 39 years old, the age of the remaining 22 subjects at the time of operation for CMT ranged from 5 months old to 12 years old with the mean age being 50.82 ± 97.63 months old. All the subjects were born at full term with normal birth weight. The method of child birth was vaginal delivery for 18 subjects and Cesarean section for 5 subjects. Three subjects (subject#5 in the microarray study, subjects #1 and #4 in the QRT-PCR study) did not receive any physical therapy for CMT, which might lower the concern on any possible influence of the physical therapy on the gene expression of the CMT. The subject #5 in the microarray study suffered from CMT since his birth without having any physical therapy for CMT. He underwent the surgery at the age of 39 years old.

### The differentially expressed genes of CMT

Overall, 33,297 probes covering 20,435 genes was significantly expressed from microarray gene expression analysis. Among the 20,435 genes, 269 genes (1.32%) were identified as DEGs. 165 genes (61.34%) were over expressed and 104 genes (38.66%) were down expressed in the T-CMT, compared to those in the T-control. The top-20 over-expressed genes based on their fold changes are listed in Table 1. *EGF-like repeats and discoidin I-like 3 *(*EDIL3*; fold change = 9.852), *asporin *(*ASPN*; 8.240), *thrombospondin 4 *(*THBS4*; 8.197), *tenomodulin *(*TNMD*; 7.576) and *nephroblastoma *(*NOV*; 5.583) were the 5 most over expressed genes in the T-CMT (Table 1). Among the 104 down-expressed genes, *aquaporin 4 *(*AQP4*) was mostly significant (fold change = -9.814; Table S4 in Additional file 1).

**Table 1:** The top-20 over-expressed genes (sorted according to the fold changes).

Gene symbol	Full name of the gene	Gene ID	Fold change of the expression level	*p*-value
***EDIL3***	***EGF-like repeats and discoidin I-like domains 3***	**10085**	**9.852**	**0.01**
*ASPN*	*asporin*	54829	8.240	0.002
***THBS4***	***thrombospondin 4***	**7060**	**8.197**	**0.004**
*TNMD*	*tenomodulin*	64102	7.576	0.047
*NOV*	*nephroblastoma overexpressed gene*	4856	5.583	0.002
*SFRP2*	*secreted frizzled-related protein 2*	395546	4.564	0.021
***SFRP4***	***secreted frizzled-related protein 4***	**6424**	**4.534**	**<0.001**
*MXRA5*	*matrix-remodelling associated 5*	25878	4.506	0.019
***FMOD***	***fibromodulin***	**2331**	**4.252**	**0.014**
***CTSK***	***cathepsin K***	**1513**	**4.099**	**0.001**
***COL14A1***	***collagen, type XIV, alpha 1***	**7373**	**4.022**	**0.001**
***LOX***	***lysyl oxidase***	**153455**	**4.010**	**<0.001**
*FAM38B*	*family with sequence similarity 38, member B*	63895	4.000	0.016
*BGN*	*biglycan*	633	3.764	<0.001
*GXYLT2*	*glucoside xylosyltransferase 2*	727936	3.604	0.01
*FIBIN*	*fin bud initiation factor homolog*	387758	3.547	<0.001
*STEAP2*	*six transmembrane epithelial antigen of the prostate 2*	261729	3.471	0.001
*LUM*	*lumican*	4060	3.440	<0.001
*DPT*	*dermatopontin*	1805	3.401	0.003
*THY1*	*Thy-1 cell surface antigen*	7070	3.390	0.002

Bolds are the DEGs which were double confirmed by QRT-PCR study.

### Clustering and principal component analyses of expression levels of DEGs distinguished CMT from non-CMT tissue

**Figure 2:**
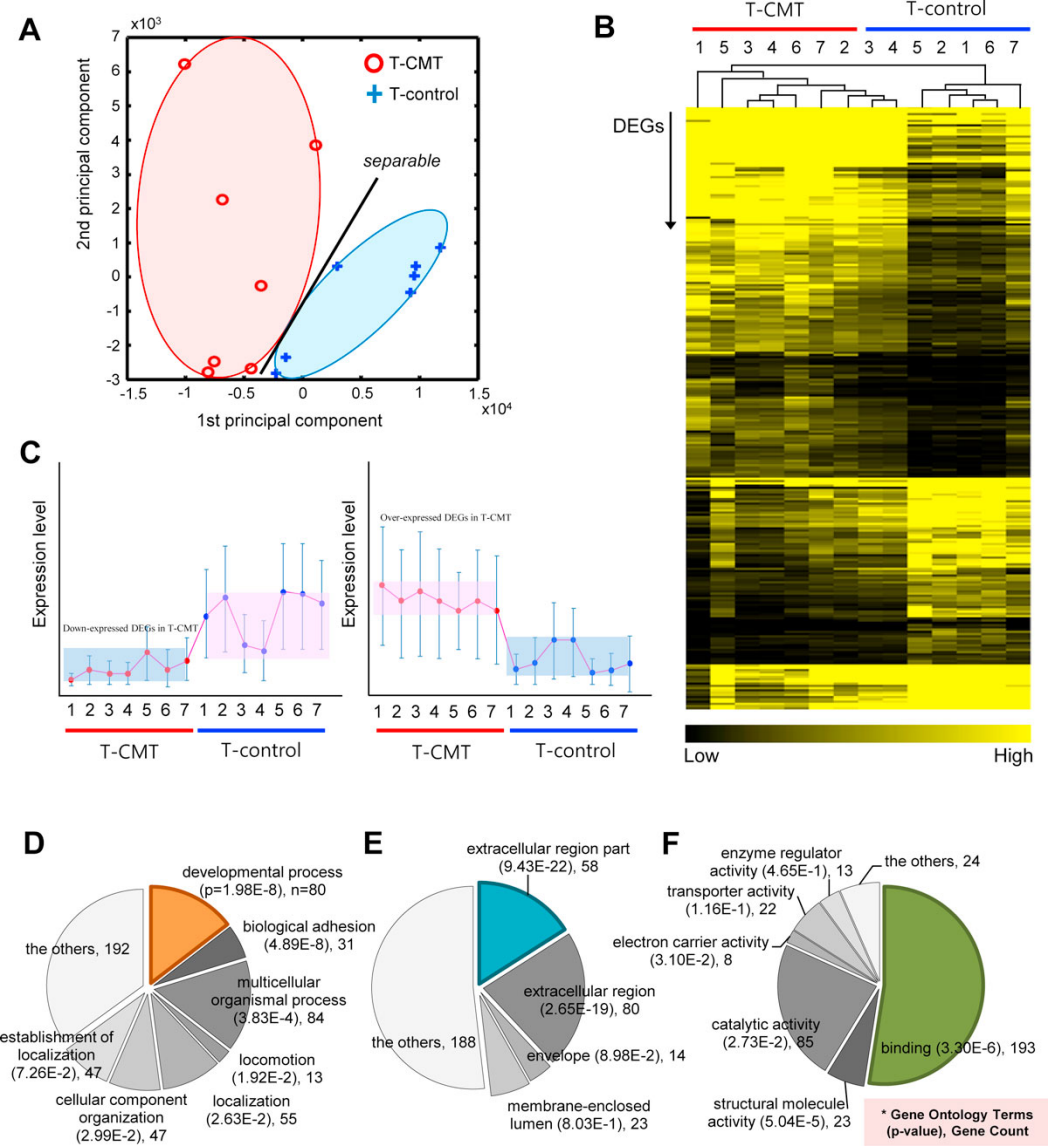
**Discriminant powers of the DEGs between T-CMT and T-control and gene ontology enrichment analysis**. The result of principal component analysis (PCA) showing a visible separation of the expression level of 269 DEGs between the T-CMT and the T-control (A). The hierarchical congregating method of the heat map shows the distinct differences of expression levels of the 269 DEGs between the T-CMT and the T-control (B). The result of k-means clustering method showing that DEGs can be divided into two groups according to their expression pattern (C). The pie diagram showing the GO enrichment analysis in terms of the biological processes, the cellular components, and molecular functions of the 269 DEGs of CMT. "n" indicates the number of genes having a corresponding GO term and "p" indicates its *p*-value (D-F).

The 269 DEGs showed a clear separation between the T-CMT and the T-control in the two dominant PCA components (Figure 2A). The heat map of the hierarchical clustering method also showed that the gene expression levels of all the DEGs were quite different between the T-CMT and the T-control (Figure 2B). Finally, we clustered DEGs with a k-means clustering algorithm according to their expressions (Figure 2C), showing that the DEGs can be divided into two groups according to their expression pattern. Taken together, these PCA and clustering results imply that the expression patterns of DEGs are distinct and discriminative according to the T-CMT or the T-control.

### Gene ontology enrichment analysis of DEGs revealed dysregulated functions in CMT

Next, the gene ontology (GO) enrichment analysis was conducted to define the dysregulated functional categories of the DEGs in terms of the biological processes, the cellular components and the molecular functions. The results of GO enrichment analysis of the 269 DEGs of CMT are illustrated in the pie diagrams (Figure 2D-F). In terms of biological processes (Figure 2D), the most significant functional term was 'developmental process', where 80 genes among the selected 269 DEGs were related to the term (*p*-value = 1.98E-8). Moreover, 'biological adhesion' (*p*-value = 4.89E-8, 31 genes) and 'multicellular organismal process' (*p*-value = 3.83E-4, 84 genes) were also significantly enriched. In terms of the cellular components (Figure 2E), 'extracellular region part' (*p*-value = 9.43E-22, 58 genes) and 'extracellular region' (*p*-value = 2.65E-19, 80 genes) were most enriched in the DEGs of CMT. In relation to the molecular function (Figure 2F), the significant functional categories of the DEGs were 'binding' (*p*-value = 3.30E-6, 193 genes) and 'structure molecule activity' (*p*-value = 3.83E-4, 84 genes). The meaningful subordinate concepts of the functional categories are shown in Figure S2 in Additional file 1. In summary, the terms related to developmental process, extracellular region, and binding were significantly dysregulated functions in the DEGs of CMT.

### Protein network analysis of differentially expressed genes indicated CMT-related functional modules

**Figure 3:**
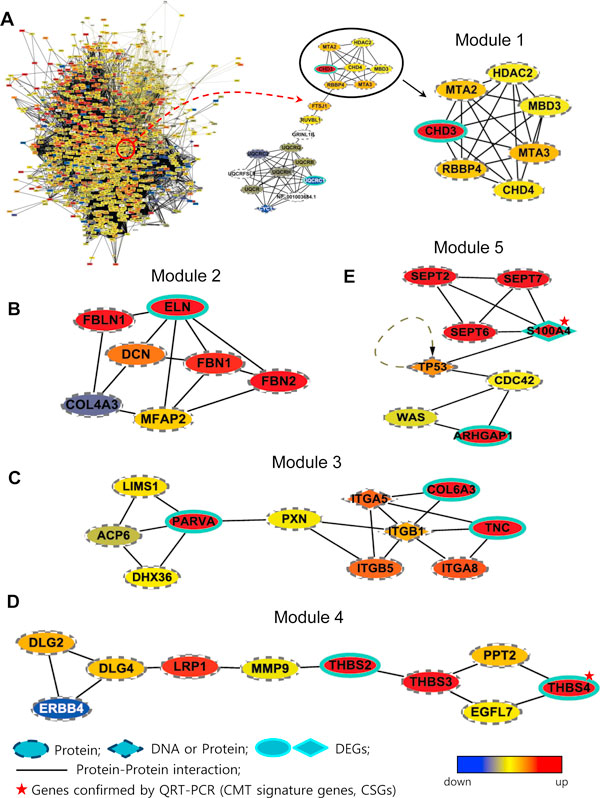
**Five CMT-related network modules**. The solid lines indicate the interactions between protein and protein. The oval shaped nodes mean proteins and the diamond shaped nodes represent proteins or DNAs depending on their role in interactions. The nodes with a highlighted border are DEGs or the proteins encoded by DEGs. The node color means the degree of the expression level of a gene in T-CMT as compared to that of T-control, where red means over-expression and blue means down-expression in the T-CMT. (A) The process of finding CMT-related network modules. The most over expressed protein in this module was the chromodomain helicase DNA-binding protein 3 (CHD3). CHD3 is one of the core subunits of Mi-2/NuRD complexes which is a novel regulator of DNA damage responses. (B) A modules showing proteins such as elastin (ELN), fibrilin 1 (FBN1) and fibrilin 2 (FBN2), which are related with elastic fiber formation. (C) A module showing over-expression of collagen fibrillogenesis, including tenacin (TNC), alpha-parvin (PARVA) and Type VI collagen A3. TNC is an extracellular protein that functions as an organizer of collagen fibrillogenesis. (D) A module showing that thrombospodins (THBS), which are regulators of collagen fibrillongenesis, are over-expressed. (E) A module showing microtubular dimerization, representing cytoskeletal rearrangement. S100A4 and Rho GTPase activating protein 1 (ARHGAP1) help microtubule to depolymerize if there is cellular strain, while septin 2, 6 and 7 work together to maintain microtubular polymerization.

The number of modules with more than 1 MCODE score was 8. Among them, 5 modules included at least one protein encoded by DEG and another protein their association with CMT was previously known. These 5 modules were selected as CMT-related protein network modules and they have densely known protein interactions among the identified DEGs (Figure 3). The module 1 shown in Figure 3A was related to the over-expression of the Mi-2/nucleosome remodeling and the histone deacetylation (NuRD) complex which is known to a regulator of DNA damage responses [22]. The clinical implication of this module is that there might be biological responses to DNA damage within the CMT tissue. In the network module 2 (Figure 3B), the proteins related to elastic fiber formation such as elastin (ELN), fibrilin 1 (FBN1) and fibrilin 2 (FBN2) were up-regulated in CMT. Elastic fiber is composed of FBN and ELN where the FBNs appear to provide a scaffold for the deposition of ELN. Moreover, module 3 (Figure 3C) represents over-expression of collagen fibrillogenesis including tenacin (TNC) which is an extracellular protein functioning as an organizer of collagen fibrillogenesis. Alpha-parvin is a protein that is encoded by parvin alpha (*PARVA*) in humans. Members of the parvin family, including PARVA, are actin-binding proteins. The network module 4 (Figure 3D) was related to the collagen fibrillogenesis, where thrombospondins (THBS) which is known to play a regulator in collagen fibrillogenesis was one of mostly over expressed genes. Lastly, the network module 5 (Figure 3E) was related to microtubular dimerization, representing cytoskeletal rearrangement related with mechanical strain, especially regarding microtubule dynamic instability. S100A4 and Rho GTPase activating protein 1 (ARHGAP1) help microtubule to depolymerize if there is cellular strain, while septin 2, 6, 7 work together to maintain microtubular polymerization.

In network ontology analysis, we can confirm our findings in protein network analysis that module 4 was related to the collagen fibrillogenesis and module 5 to cytoskeletal part (Table 2). There was no GO term of which p-value was less than 0.1 in module 1.

**Table 2:** Gene ontology terms of CMT-related functional modules revealed by network ontology analysis.

Module	GO domain	GO term	Related interaction	p-value
Module 2	BP	developmental process	DCN-ELN, ELN-FBN1, ELN-FBN2, DCN-FBN1, FBN1-FBN2	0.0839

Module 3	BP	cell division	SEPT2-SEPT7, SEPT2-SEPT6, SEPT6-SEPT7	0.0503
	BP	cell cycle	SEPT2-SEPT7, SEPT2-SEPT6, SEPT6-SEPT7	0.0503
	CC	septin complex	SEPT2-SEPT7, SEPT2-SEPT6, SEPT6-SEPT7	0.0503
	CC	cell cortex part	SEPT2-SEPT7, SEPT2-SEPT6, SEPT6-SEPT7	0.0503

Module 4	BP	signaling	ITGB1-ITGB5, ITGA5-ITGB1, ITGB1-TNC, ITGA8-ITGB1, ITGA5-ITGB5, ITGA5-TNC, ITGA8-TNC	0.0091
	BP	response to stimulus	ITGB1-PXN, ITGA5-ITGB1, ITGB1-TNC, ITGA8-ITGB1, ITGA5-TNC, ITGA8-TNC	0.0521
	BP	biological adhesion	PARVA-PXN, ITGB1-PXN, ITGB5-PXN, ITGB1-ITGB5, COL6A3-ITGB1, ITGA5-ITGB1, ITGB1-TNC, ITGA8-ITGB1, ITGA5-ITGB5, COL6A3-ITGA5, ITGA5-TNC, ITGA8-TNC	0.0768
	BP	cell adhesion	PARVA-PXN, ITGB1-PXN, ITGB5-PXN, ITGB1-ITGB5, COL6A3-ITGB1, ITGA5-ITGB1, ITGB1-TNC, ITGA8-ITGB1, ITGA5-ITGB5, COL6A3-ITGA5, ITGA5-TNC, ITGA8-TNC	0.0768
	BP	signaling pathway	ITGB1-ITGB5, ITGA5-ITGB1, ITGA8-ITGB1, ITGA5-ITGB5	0.0845
	BP	cell-substrate adhesion	ITGB1-PXN, ITGB5-PXN, ITGB1-ITGB5, ITGA8-ITGB1	0.0845
	BP	cell-matrix adhesion	ITGB1-PXN, ITGB5-PXN, ITGB1-ITGB5, ITGA8-ITGB1	0.0845
	BP	cell surface receptor linked signaling pathway	ITGB1-ITGB5, ITGA5-ITGB1, ITGA8-ITGB1, ITGA5-ITGB5	0.0845
	BP	integrin-mediated signaling pathway	ITGB1-ITGB5, ITGA5-ITGB1, ITGA8-ITGB1, ITGA5-ITGB5	0.0845
	CC	integral to membrane	ITGB1-ITGB5, ITGA5-ITGB1, ITGA8-ITGB1, ITGA5-ITGB5	0.0621
	CC	intrinsic to membrane	ITGB1-ITGB5, ITGA5-ITGB1, ITGA8-ITGB1, ITGA5-ITGB5	0.0621
	CC	receptor complex	ITGB1-ITGB5, ITGA5-ITGB1, ITGA8-ITGB1, ITGA5-ITGB5	0.0621
	CC	integrin complex	ITGB1-ITGB5, ITGA5-ITGB1, ITGA8-ITGB1, ITGA5-ITGB5	0.0621
	MF	signal transducer activity	ITGB1-ITGB5, ITGA5-ITGB1, ITGA8-ITGB1, ITGA5-ITGB5	0.0621
	MF	receptor activity	ITGB1-ITGB5, ITGA5-ITGB1, ITGA8-ITGB1, ITGA5-ITGB5	0.0621
	MF	molecular transducer activity	ITGB1-ITGB5, ITGA5-ITGB1, ITGA8-ITGB1, ITGA5-ITGB5	0.0621

Module 5	CC	postsynaptic density	DLG2-ERBB4, DLG2-DLG4, DLG4-ERBB4	0.0116
	CC	cytoskeletal part	DLG2-ERBB4, DLG2-DLG4, DLG4-ERBB4	0.0116
	CC	synapse part	DLG2-ERBB4, DLG2-DLG4, DLG4-ERBB4	0.0116

Only GO terms of which p-values were less than 0.1 were listed. GO, Gene Ontology; BP, biological process; MF, molecular function; CC, cellular component

### QRT-PCR confirmed the differential expression of the CMT-gene signature

**Figure 4:**
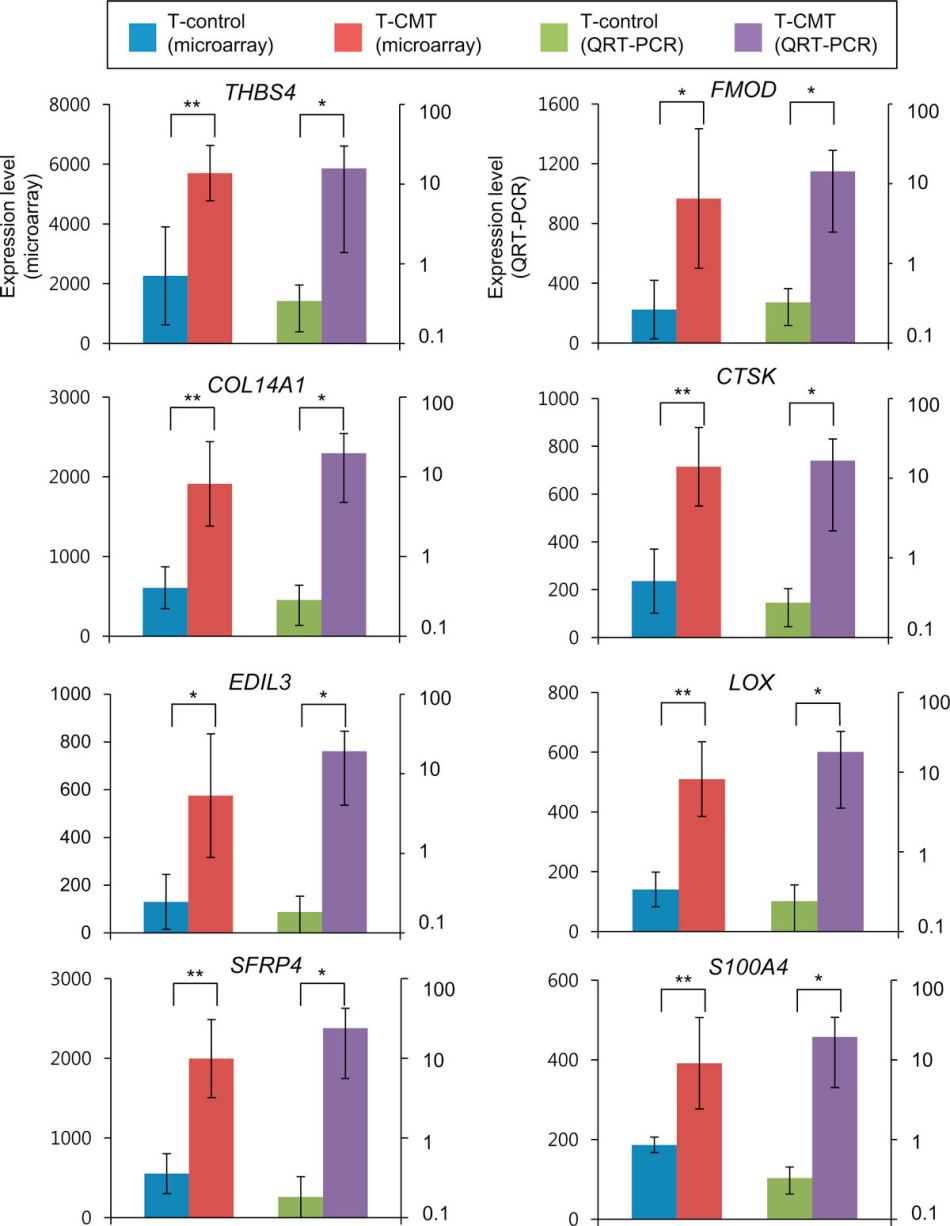
**Validation of the differentially expressed genes by QRT-PCR**. Average expression levels of validated 8 genes are shown with their 95% confidence interval. The first to fourth bar means average expression level of T-control and T-CMT from microarray and average level of T-control and T-CMT from QRT-PCR, respectively. *p < 0.05, **p < 0.01

These expression levels of the 8 genes from the QRT-PCR were well correlated with those from microarray analysis even though the samples are independent between them (0.41 of R^2^, Figure 4 and S3 in Additional file 1). We called the eight confirmed genes as "CMT signature genes (CSGs)" in the further analysis.

### The gene signature of CMT highly correlated with radiological severity of CMT

**Figure 5:**
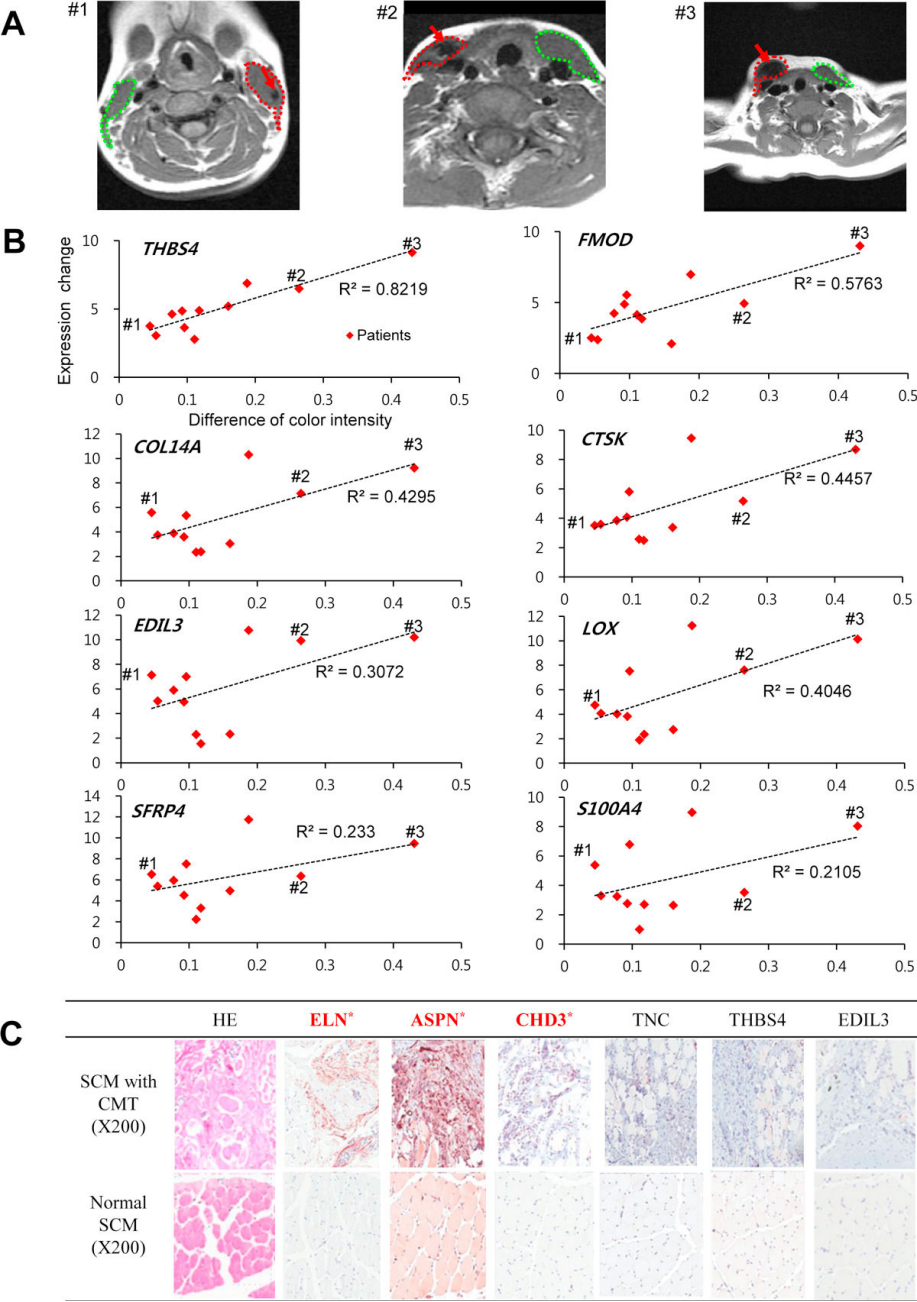
**Correlation between the QRT-PCR findings and the MRI findings and immumohistochemical staining showing the expression of the proteins encoded by the DEGs of CMT (X200)**. The MRI of subjects #1, #2 and #3. Red and green circles indicate the cross sectional areas of the SCM with or without CMT, respectively. The red arrows indicate the lowest signal areas of CMT. (A). The correlation between the differences of MRI color intensities and the differences of expressions of 8 DEGs in the tissue with and without CMT for a total of 11 independent patients (B). Immunohistochemical staining shows significantly increased immuno-expression of ELN, ASPN and CHD3 (C).

We examined relationship between the gene expression levels of CSGs and the radiological severity of CMT. The MRIs of 11 subjects were shown in Figure 5A and S4 in Additional file 1, where red and green circles indicate the cross sectional areas of the SCM with and without CMT, respectively. The red arrows indicate the lowest signal area of CMT. We observed that the expression changes of CSGs from QRT-PCR were well correlated with the intensity difference between the SCM-CMT and the SCM-control on MRI images (R^2^ = 0.82~0.21; Figure 5B). In the uppermost left graph of Figure 5B, for example, *THBS4 *showed a high correlation between the fold change of the expression level and difference in color intensity of the tissues with/without CMT (R^2^ = 0.82). The subject #3 with the highest degree of *THBS4 *over-expression in the tissue with CMT showed the biggest difference in the grey intensities of MRI images of SCM with/without torticollis. On the other hand, the subject #1, who has smallest difference in the *THBS4 *expression level, showed the lowest degree of difference in grey color intensity in its MRI images. This also indicates that the subjects who showed lower signal intensity on the pre-operational T1-weighted MRI tended to show higher expression levels of *THBS4 *as compared to that of the T-control. Similar correlations can be observed for the seven other genes, including *FMOD *(R^2^ = 0.5763), *COL14A1 *(R^2 ^= 0.4295), *CTSK *(R^2 ^= 0.4457), *EDIL3 *(R^2 ^= 0.3072), *LOX *(R^2 ^= 0.4046), *SFRP4 *(R^2 ^= 0.233), and *S100A4 *(R^2 ^= 0.2105). Therefore, the expression level changes of the CSGs were well correlated with the difference in grey color intensity of SCM with/without CMT indicating the clinical severity of CMT.

### Expression of the proteins encoded by the differentially expressed genes of CMT

Expression of 6 proteins encoded by the DEGs of CMT was examined by a semi-quantitation of IHC assay (Figure 5C, Table S5 in Additional file 1). IHC examination showed significantly increased immune-expression of ELN, ASPN, and CHD3 encoded by the 3 DEGs including *ELN, ASPN, and CHD3 *compared to the normal SCM (*p*< 0.05). However, the proteins such as TNC, THBS4 and EDIL3 did not show high increased expression in the CMT tissue.

## Discussion

This research provides an integrative analysis of gene expression profiling with clinical severity of CMT and the expression of proteins encoded by DEG. Furthermore, the CMT-related network modules were identified, which provided more in-depth understanding of pathophysiology of CMT. The differentially expressed genes of CMT were first identified from microarray analysis, where the main deregulated function of the DEGs was related to the extracellular region part during the developmental processes. The gene expression signatures of CMT was first characterized by the over-expression of collagen and elastin fibrillogenesis along with the evidence of DNA repair mechanism and the cytoskeletal rearrangement possibly related with mechanical damage. Furthermore, the pre-operational MRI images of CMT were shown to be correlated well with the gene expression signature of CMT with 8 significant DEGs. The protein expressions of ELN, ASPN and CHD3 which were encoded by the meaningful DEGs of CMT, also confirmed the different gene expression between the CMT and normal SCM. To the best of our knowledge, this is the first report that has examined the transcriptome of human CMT tissue, co-analyzing the data of the tissue with and without CMT from the same subjects.

One of the most interesting findings of this study was that the mRNA expression levels of the CSGs correlated well with the radiological severity of CMT in terms with the pre-operational MRI findings of CMT. For example, *THBS4 *showed the highest correlation with the neck MRI findings as seen in Figure 5B. Thrombospondins (THBSs) are extracellular modular glycoproteins that function as regulators of collagen fibrillogenesis [23,24]. While *THBS4 *was one of the top-10 over-expressed genes in CMT in this study (Table 1), *thrombospondin 5 *(also known as *cartilage oligomeric matrix protein*; *COMP*) was also over expressed DEGs in CMT among 5 mammalian *THBS1*~*THBS5*. While the biological function of THSB3 and THSB4 has not been clearly known, THBS4 expression is highly induced at the neuromuscular junction following injury [25]. It is also expressed in tendon where it can form mixed pentamers with THBS5 [25-27]. Furthermore, expressions of the three genes including *CHD3*, *ELN*, and *asporin*, show same correlation between T-CMT and T-control as expressions of proteins encoded by them. Among the CSGs, however, the mRNA expressions of three genes including *THBS4*, *EDIL3 *and *TNC*, were not coincident with those of protein. It might be caused by post-transcriptional and/or post-translational regulations. For example, a microRNA (miRNA) as a post-transcriptional regulator degrades or silences target mRNAs by binding to complementary sequences on them [28]. Further studies are needed to discover which biological processes are associated with unmatched expression between genes and proteins in CMT and how they affect the pathogenesis of CMT.

The five CMT-related network modules indicate that one of the critical pathways to the pathogenesis of CMT is fibrosis related with collagen and elastin fibrillogenesis with the evidence of DNA repair mechanism and cytoskeletal rearrangement possibly related with mechanical strain. The gene for CHD3 was the most over-expressed in our study among the subunits of the Mi-2/NuRD complex (Figure 3A). The complex Mi-2/NuRD is the only known protein entity that uniquely possesses both nucleosome remodeling and histone deacetylase activities and it is a novel regulator of DNA damage responses [22,29]. We also found fibrosis-related pathways in CMT. ELN was differentially over-expressed in CMT along with other genes that are related with elastic fiber formation such as FBN [30]. Figure 3E shows a module representing cytoskeletal rearrangement related with mechanical strain, especially regarding microtubule dynamic instability. Microtubule is a polymeric tubulin which is responsible for cellular integrity as one of cytoskeletons. When cells experience cellular strain, microtubule tends to depolymerize, ending up with the form of dimer.

This study has several limitations to be considered. First, the differentiation of tissue with CMT and the tissue without CMT from the myectomized tissue was done by the gross appearance of the tissue. From the tissue that was myectomized, the tissue that showed a tendon-like appearance of white- or pale-pinkish color was considered tissue with CMT and the tissue that was at the periphery of CMT tissue and it looked like normal pinkish muscle was considered tissue without CMT. Four subjects, who did not show T-control within the myectomized tissue, were not able to be enrolled in this study. The heat map of the hierarchical clustering method also showed that 7 samples of T-CMT (#1-7) and 2 samples of T-control (#3, #4) showed similar expression levels compared to the remaining 5 samples of T-control (Figure 2B). The histopathologic findings of the subject #3 and #4 in the microarray study showed severe fibrosis, which might explain relatively little amount of T-control. Therefore, contamination of T-CMT into the T-control could be a possible explanation for this phenomenon. Second, most of the subjects had physical therapy, including ultrasound. However, there were 3 subjects (subject#5 in the microarray study, subjects #1 and #4 in the QRT-PCR study) who did not receive any physical therapy at all and the gene expression signature of these subjects showed similar pattern with the other subjects. Therefore, it is unlikely that the gene expression profiling could be modified by this intervention. Third, the gene expression signature of CMT shown in this study is a cross-sectional finding of the intermediate or advanced stage of CMT. Therefore, we do not know the acute response to CMT.

## Conclusions

In conclusion, we were able to provide an integrative analysis of CMT from gene expression to clinical indication. The CMT-related network modules gave us more in-depth understanding of pathophysiology of CMT. Based on the results of this study, CMT might be defined as a developmental disorder of the SCM characterized by fibrosis, ending up with the shortening of the SCM.

## Competing interests

The authors declare that they have no competing interests.

## Authors' contributions

SYY, DY, MAL, RWP, KL and JJ designed research; SYY enrolled subjects; MCP, IJL, JDL, JHL and EYS participated in the collection of specimens; YIN and JJ prepared the genomic DNA and total RNA and carried out the real-time PCR assay and immunohistochemistry assay; SYY, DY, JHK, KSK and KL analyzed data; SYY, DY and KL wrote the paper; All authors read and approved the final manuscript.

## Supplementary Material

Additional file 1 **Supplementary tables and figures**. Supplementary table S1-5 and supplementary figure S1-4 were included in this additional file. Click here for file

## Declarations

The publication costs for this article were funded by the corresponding author.

This article has been published as part of *BMC Medical Genomics *Volume 6 Supplement 2, 2013: Selected articles from the Second Annual Translational Bioinformatics Conference (TBC 2012). The full contents of the supplement are available online at http://www.biomedcentral.com/bmcmedgenomics/supplements/6/S2.
