# Supplementary material for: Integrative analysis of congenital muscular torticollis: from gene expression to clinical significance
Source: BMC Med Genomics. 2013 May 7;6(Suppl 2):S10. doi: 10.1186/1755-8794-6-S2-S10 (PMC3654872; doi:10.1186/1755-8794-6-S2-S10)
Supplement: Additional file 1 — Supplementary tables and figures. Supplementary table S1-5 and supplementary figure S1-4 were included in this additional file. [file 1755-8794-6-S2-S10-S1.doc]

**SUPPLEMENTARY TABLES AND FIGURES**

Integrative Analysis of Congenital Muscular Torticollis: from Gene Expression to Clinical Significance

Shin-Young Yim, MD, PhD^1,†^, Dukyong Yoon, MD, MS^2,†^, Myong Chul Park, MD, PhD^3^, Il Jae Lee, MD, PhD^3^, Jang-Hee Kim, MD, MS^4^, Myung Ae Lee,PhD^5^, Kyu-Sung Kwack, MD, PhD^6^, Jan-Dee Lee, MD, PhD^7^, Jeong-Hun Lee, MD^8^,  Euy-Young Soh, MD, PhD^8^, Young-In Na, MS^9^, Rae Woong Park, MD, PhD^2^, KiYoung Lee, PhD^2,*^ and Jae-Bum Jun, MD, PhD^9,*^

^1^The Center for Torticollis, Department of Physical Medicine and Rehabilitation, Ajou University School of Medicine, Suwon, Republic of Korea; ^2^Department of Biomedical Informatics, Ajou University School of Medicine, Suwon, Republic of Korea; ^3^Department of Plastic and Reconstructive Surgery, Ajou University School of Medicine, Suwon, Republic of Korea; ^4^Department of Pathology, Ajou University School of Medicine, Suwon, Republic of Korea;^5^Brain Disease Research Center, Ajou University School of Medicine, Suwon, Republic of Korea; ^6^Department of Radiology, Ajou University School of Medicine, Suwon, Republic of Korea; ^7^Department of Surgery, Yonsei University College of Medicine, Seoul, Republic of Korea;^8^Department of Surgery, Ajou University School of Medicine, Suwon, Republic of Korea; ^9^Department of Rheumatology, The Hospital for Rheumatic Diseases, Hanyang University College of Medicine, Seoul, Republic of Korea

**Table S1** The genes and their primers used in the quantitative real-time PCR analyses.

| Gene symbol | Full name | Gene ID | Primer sequence |
| --- | --- | --- | --- |
|  |  |  |  |
| *THBS4* | *Thrombospondin 4* | 7060 | Fw : gacaatgatggtatcccagacc  Rv : ctcacagatgtctcccactcc |
|  |  |  | Fw : aggtggattccgttcacaat  Rv : cagctcttccaagaagtcctgt |
| *FMOD* | *Fibromodulin* | 2331 | Fw : gcacttaattcttcccatttcaaa  Rv : ggctaactctcctccaaca |
|  |  |  | Fw : caaccagctgcagaagatcc  Rv : gcagaagctgctgatggag |
| *COL14A1* | *Collagen, type XIV, alpha 1* | 7373 | Fw : aggtcatctgggggttcct  Rv : tgaactctggctgatctgga |
|  |  |  | Fw : aactgatggaagatcacaagatga  Rv : cacaccaattgcaaaaatgc |
| *CTSK* | *Cathepsin K* | 1513 | Fw : tctctgtggccattgatgc  Rv : gcagctttcatcataatacacacc |
|  |  |  | Fw : aacgaagccagacaacag  Rv : tccagtatctcctcagggta |
| *EDIL3* | *EGF-like repeats and discoidin I-like domains 3* | 10085 | Fw : ttcttggctgtgaactgtcg  Rv : ccatgttgagcgttctgaag |
|  |  |  | Fw : gggattcactgtcagcacaa  Rv: ctcacaggaatagttagcaacaaga |
| *LOX* | *Lysyl oxidase* | 153455 | Fw : tctctttgaaatctcataggatagtc  Rv : aactctcagtgccaggaa |
|  |  |  | Fw : cctggttcctgaatctgac  Rv : ttgccttctaatacggtgaa |
| *SFRP4* | *Secreted frizzled-related protein 4* | 6424 | Fw : ttagttggctaatgctcaagtatt  Rv : gtcaaattattctcagggtggat |
|  |  |  | Fw : gtcccgctcattacaaattctt  Rv : agcaattttcaagaagcatcatc |
| *S100A4* | *S100 calcium binding protein A4* | 6275 | Fw : agggtgacaagttcaagctca  Rv : aagcagcttcatctgtcctttt |
| *GAPDH* | *Glyceraldehyde-3-phosphate dehydrogenase* | 2597 | Fw : tcgacagtcagccgcatcttcttt  Rv : gcccaatacgaccaaatccgttga |

Fw: forward, Rv: reverse

**Table S2.** Specifications of antibodies used for immunohistochemistry.

| Antibody | Cat. no | Source | Antibody dilution | Antigen retrieval | Antibody incubation |
| --- | --- | --- | --- | --- | --- |
| Elastin (ELN) | ab9519 | Abcam,Cambridge, MA | 1:50 | Proteinase K | 24 hours at 4℃ |
| Asporin (ASPN) | ab58741 | Abcam,Cambridge, MA | 1:50 | HIER in citrate buffer | 24 hours at 4℃ |
| Chromodomain helicase DNA-binding protein 3 (CHD3). | ab109195 | Abcam,Cambridge, MA | 1:100 | HIER in citrate buffer | 1 hour at RT |
| Tenascin (TNC) | ab82449 | Abcam,Cambridge, MA | 1:100 | HIER in citrate buffer | 24 hours at 4℃ |
| Thrombospondin 4 (THBS4) | sc-20646 | Santa Cruz Biotechnology, Santa Cruz, CA | 1:50 | HIER^*^ in citrate buffer | 24 hours at 4℃ |
| EGF-like repeats and discoidin I-like domains 3 (EDIL3) | 12580-1-AP | PTGlab, Chicago, IL | 1:100 | HIER in citrate buffer | 1 hour at RT^**^ |

^*^HIER - Heat Induced Epitope Retrieval

^**^RT- Room temperature

**Table S3.** The demographic characteristics of the subjects.

| Group | No | Gender | Location of CMT | Age at the time of operation for CMT  (months old) | Gestational period (weeks) | Birth weight (gram) | Method of child birth  (V:C)^*^ | Previous physical therapy |
| --- | --- | --- | --- | --- | --- | --- | --- | --- |
| Microarray | 1 | M | Left | 26 | 40 | 3600 | V | + |
|  | 2 | M | Right | 6 | 40 | 4290 | V | + |
|  | 3 | M | Left | 7 | 40 | 3900 | V | + |
|  | 4 | M | Left | 31 | 39 | 3000 | V | + |
|  | 5 | M | Right | 476 | 40 | 3000 | V | - |
|  | 6 | M | Right | 17 | 39 | 3700 | V | + |
|  | 7 | M | Right | 5 | 37 | 3140 | C | + |
| QRT-PCR | 1 | F | Left | 48 | 41 | 3780 | V | - |
|  | 2 | M | Right | 30 | 40 | 3200 | C | + |
|  | 3 | M | Right | 11 | 39 | 3200 | V | + |
|  | 4 | F | Right | 101 | 38 | 2900 | C | - |
|  | 5 | M | Left | 78 | 40 | 3750 | V | + |
|  | 6 | F | Left | 27 | 38 | 3150 | V | + |
|  | 7 | M | Right | 58 | 41 | 4500 | V | + |
|  | 8 | M | Right | 120 | 40 | 3200 | C | + |
|  | 9 | M | Left | 31 | 38 | 3000 | V | + |
|  | 10 | M | Right | 20 | 40 | 3200 | V | + |
|  | 11 | M | Right | 17 | 40 | 4080 | V | + |
| IHC | 1 | M | Right | 11 | 38 | 3060 | C | + |
|  | 2 | M | Right | 11 | 40 | 3110 | V | + |
|  | 3 | F | Left | 11 | 38 | 3900 | V | + |
|  | 4 | M | Right | 15 | 40 | 3120 | V | + |
|  | 5 | M | Right | 12 | 40 | 3610 | V | + |

^*^ Vaginal delivery: Cesarean section

**Table S4** All 269 differentially expressed genes in microarray study.

| Name | Fold change |  | Name | Fold change |  | Name | Fold change |  | Name | Fold change |  | Name | Fold change |
| --- | --- | --- | --- | --- | --- | --- | --- | --- | --- | --- | --- | --- | --- |
| EDIL3 | 9.85 |  | COMP | 2.57 |  | FKBP10 | 2.19 |  | ZNF521 | 2.00 |  | PPP1R1A | -2.24 |
| ASPN | 8.24 |  | CCDC3 | 2.56 |  | GPC3 | 2.18 |  | PLXDC2 | 2.00 |  | SLC2A4 | -2.26 |
| THBS4 | 8.20 |  | CDH11 | 2.54 |  | ANXA1 | 2.18 |  | TIMP2 | 2.00 |  | RPL3L | -2.27 |
| TNMD | 7.58 |  | TIMP1 | 2.53 |  | CDO1 | 2.17 |  | GOT2 | -2.00 |  | ADHFE1 | -2.27 |
| NOV | 5.58 |  | TUBA1A | 2.53 |  | DKFZP586H2123 | 2.17 |  | TMEM16E | -2.01 |  | ECH1 | -2.27 |
| SFRP2 | 4.56 |  | MLLT11 | 2.52 |  | FSTL1 | 2.15 |  | ACADSB | -2.01 |  | FIT1 | -2.28 |
| SFRP4 | 4.53 |  | ANTXR1 | 2.51 |  | SNX7 | 2.14 |  | DLK1 | -2.01 |  | HRASLS | -2.29 |
| MXRA5 | 4.51 |  | MGP | 2.51 |  | MAP1B | 2.14 |  | ADSSL1 | -2.03 |  | IDH2 | -2.33 |
| FMOD | 4.25 |  | ALDH1L2 | 2.47 |  | CRISPLD1 | 2.13 |  | UQCRC1 | -2.03 |  | RORC | -2.33 |
| CTSK | 4.10 |  | SERPINE2 | 2.46 |  | CYR61 | 2.13 |  | FBXO32 | -2.03 |  | ALDH1L1 | -2.33 |
| COL14A1 | 4.02 |  | GOLM1 | 2.46 |  | PTGFRN | 2.13 |  | BRP44L | -2.03 |  | HADHB | -2.34 |
| LOX | 4.01 |  | CD44 | 2.45 |  | CHODL | 2.13 |  | LOC729830 | -2.03 |  | ASB2 | -2.34 |
| FAM38B | 4.00 |  | OMD | 2.45 |  | COBLL1 | 2.12 |  | TYRP1 | -2.04 |  | ZADH1 | -2.34 |
| BGN | 3.76 |  | MAP1A | 2.44 |  | F2R | 2.12 |  | LRRTM4 | -2.05 |  | MAP2K6 | -2.36 |
| GLT8D4 | 3.60 |  | TRIM38 | 2.43 |  | PYGL | 2.12 |  | OSBPL11 | -2.05 |  | MPP7 | -2.41 |
| FIBIN | 3.55 |  | ASAM | 2.43 |  | CHD3 | 2.12 |  | CD38 | -2.05 |  | CMBL | -2.42 |
| STEAP2 | 3.47 |  | BOC | 2.42 |  | CTSO | 2.12 |  | SLC7A2 | -2.06 |  | LPL | -2.44 |
| LUM | 3.44 |  | ADIPOQ | 2.41 |  | KIAA1598 | 2.11 |  | TMEM159 | -2.06 |  | HSDL2 | -2.44 |
| DPT | 3.40 |  | SPON1 | 2.41 |  | DAB2 | 2.11 |  | NT5C1A | -2.07 |  | ETFDH | -2.44 |
| THY1 | 3.39 |  | CCDC80 | 2.41 |  | S100A4 | 2.10 |  | DLAT | -2.07 |  | ITGB6 | -2.45 |
| LOC493869 | 3.38 |  | THBS2 | 2.40 |  | PLEKHA5 | 2.10 |  | DDIT4L | -2.07 |  | ASB15 | -2.46 |
| VCAN | 3.31 |  | HMCN1 | 2.39 |  | MYH10 | 2.10 |  | GADL1 | -2.08 |  | ASB4 | -2.49 |
| COL12A1 | 3.30 |  | PTPN13 | 2.38 |  | ANXA2 | 2.09 |  | LPIN1 | -2.08 |  | PPP1R3A | -2.51 |
| CTGF | 3.21 |  | MMP2 | 2.38 |  | CCL2 | 2.08 |  | LMOD1 | -2.08 |  | C21ORF82 | -2.51 |
| FNDC1 | 3.19 |  | TNC | 2.38 |  | CHRDL1 | 2.08 |  | COX10 | -2.09 |  | ASB14 | -2.52 |
| RAB31 | 3.09 |  | CCND2 | 2.37 |  | ANXA2P2 | 2.08 |  | FASTKD1 | -2.09 |  | OCC-1 | -2.55 |
| AEBP1 | 3.07 |  | COL1A1 | 2.34 |  | TSHZ2 | 2.08 |  | TUBA8 | -2.09 |  | LOC121952 | -2.59 |
| SLC41A2 | 3.06 |  | LIMA1 | 2.34 |  | LOC652811 | 2.07 |  | LRRC2 | -2.10 |  | PHKA1 | -2.66 |
| ACTC1 | 3.05 |  | ANGPTL2 | 2.34 |  | EMP1 | 2.07 |  | COQ10A | -2.10 |  | SLC38A4 | -2.67 |
| ENPP1 | 3.05 |  | UNQ1940 | 2.34 |  | PARVA | 2.07 |  | ASB12 | -2.10 |  | CABC1 | -2.67 |
| OGN | 3.02 |  | F13A1 | 2.33 |  | RAB23 | 2.06 |  | APOO | -2.11 |  | MAOB | -2.69 |
| IGFN1 | 3.00 |  | RAI14 | 2.31 |  | LEPREL1 | 2.06 |  | ENO3 | -2.11 |  | SLC36A2 | -2.69 |
| XG | 3.00 |  | COL1A2 | 2.30 |  | FABP4 | 2.06 |  | SLC47A1 | -2.12 |  | GPD1L | -2.79 |
| ABI3BP | 2.93 |  | PDGFRL | 2.30 |  | SPARC | 2.05 |  | PLCL2 | -2.12 |  | PDE11A | -2.79 |
| DCLK1 | 2.86 |  | TES | 2.30 |  | GLI3 | 2.05 |  | KLHL30 | -2.13 |  | C3ORF43 | -2.88 |
| FAP | 2.86 |  | SRPX2 | 2.30 |  | ABHD2 | 2.05 |  | FNDC5 | -2.13 |  | PFKFB1 | -2.90 |
| THBS1 | 2.83 |  | GLIPR1 | 2.30 |  | KIAA1377 | 2.05 |  | NNT | -2.14 |  | ALPK2 | -2.92 |
| SERTAD4 | 2.82 |  | HTRA1 | 2.28 |  | ELN | 2.05 |  | C1ORF170 | -2.14 |  | SLC38A3 | -2.93 |
| S100A11 | 2.80 |  | SULF1 | 2.28 |  | CD109 | 2.05 |  | PYGM | -2.15 |  | CLCN1 | -3.03 |
| C18ORF30 | 2.79 |  | C1QTNF3 | 2.27 |  | CERCAM | 2.04 |  | ACO2 | -2.15 |  | PAIP2B | -3.03 |
| KDELR3 | 2.79 |  | CHAD | 2.27 |  | GEM | 2.04 |  | AGBL1 | -2.15 |  | C8ORFK36 | -3.03 |
| FRMD4B | 2.78 |  | SLIT2 | 2.26 |  | SERPINH1 | 2.04 |  | ACADM | -2.16 |  | ASB11 | -3.08 |
| MYO1D | 2.74 |  | CLIC1 | 2.26 |  | MFAP5 | 2.04 |  | PHYH | -2.16 |  | MYLK2 | -3.13 |
| DPP4 | 2.74 |  | PRELP | 2.25 |  | ARHGAP1 | 2.04 |  | SLC19A2 | -2.17 |  | ART3 | -3.15 |
| TMEM45A | 2.72 |  | FER1L3 | 2.25 |  | TNFAIP6 | 2.03 |  | PGAM2 | -2.17 |  | PEBP4 | -3.33 |
| NOX4 | 2.70 |  | CMKLR1 | 2.25 |  | LHFPL2 | 2.03 |  | KIAA0408 | -2.17 |  | GPT2 | -3.41 |
| VCAM1 | 2.70 |  | COL5A2 | 2.23 |  | MAMDC2 | 2.02 |  | CA2 | -2.19 |  | SLC25A30 | -3.44 |
| C7 | 2.68 |  | OLFML1 | 2.22 |  | PLOD2 | 2.02 |  | ESRRG | -2.19 |  | DHRS7C | -3.47 |
| CRABP2 | 2.64 |  | PDGFD | 2.22 |  | PDE1A | 2.02 |  | ACSS2 | -2.19 |  | MYLK3 | -3.79 |
| FN1 | 2.64 |  | HGF | 2.22 |  | LRRC17 | 2.01 |  | FABP3 | -2.20 |  | MLF1 | -4.53 |
| C18ORF58 | 2.63 |  | C9ORF19 | 2.21 |  | GJA1 | 2.01 |  | UCP3 | -2.20 |  | C8ORF22 | -5.41 |
| AHNAK2 | 2.62 |  | LTBP2 | 2.21 |  | NFATC2 | 2.01 |  | TMEM143 | -2.21 |  | NEK10 | -6.60 |
| COL6A3 | 2.57 |  | GPNMB | 2.20 |  | EMP3 | 2.01 |  | PPIF | -2.22 |  | AQP4 | -9.81 |
| GALNT5 | 2.57 |  | CAMK1D | 2.20 |  | SFRP1 | 2.00 |  | NIPSNAP3B | -2.23 |  |  |  |

***Table S5*** *Semi-quantitation of immunohistochemical study according to proteins encoded by the differentially expressed genes of CMT.*

| Proteins | Subjects with CMT | | | | | | Subjects with normal SCM | | | | | | P value |
| --- | --- | --- | --- | --- | --- | --- | --- | --- | --- | --- | --- | --- | --- |
|  | 1 | 2 | 3 | 4 | 5 |  | | 1 | 2 | 3 | 4 | 5 |  |
| Elastin (ELN) | 2 | 2 | 3 | 2 | 2 |  | | 1 | 1 | 1 | 1 | 1 | 0.008 |
| Asporin (ASPN) | 3 | 3 | 3 | 3 | 2 |  | | 1 | 1 | 2 | 1 | 1 | 0.008 |
| Chromodomain helicase DNA-binding protein 3 (CHD3). | 2 | 2 | 1 | 1 | 1 |  | | 0 | 0 | 0 | 0 | 0 | 0.008 |
| Tenascin (TNC) | 2 | 1 | 1 | 2 | 1 |  | | 1 | 1 | 1 | 1 | 1 | > 0.05 |
| Thrombospondin 4 (THBS4) | 1 | 2 | 0 | 0 | 0 |  | | 1 | 1 | 0 | 0 | 0 | > 0.05 |
| EGF-like repeats and discoidin I-like domains 3 (EDIL3) | 0 | 0 | 0 | 0 | 0 |  | | 0 | 0 | 0 | 0 | 0 | > 0.05 |

^*^*thrombospondin 4*

^**^*EGF-like repeats and discoidin I-like domains 3*

**Figure S1. The decision tree model for discriminating T-CMT and T-control**

We examined the discriminant power of the DEGs using decision tree method implemented in Clementine 12.0 (SPSS Inc., Chicago, IL, USA). 8 samples (4 T-CMT and 4 T-control) from 4 subjects were used for training data and the others for test. Decision tree models showed a mean sensitivity of 0.70 and a mean specificity of 0.82. That means that the DEGs identified from the microarray study were able to accurately differentiate the T-CMTs from the T-controls. This figure showed one example of the decision tree model. The expressed level of S100A4 in the T-CMT was greater than 225.683 in all 4 T-CMTs, while the expressed levels of S100A4 in the T-control was 225.683 or less.

**Figure S2. The Gene Ontology enrichment analysis on molecular function**

Each portion represents the Gene Ontology term (p-value) and the number of genes. (a) The significant subordinate concepts of the ‘developmental process’ were ‘anatomical structure morphogenesis’ (p-value = 7.95E−8, 48 genes), ‘skeletal system development’ (p-value = 1.45E−7, 20 genes), ‘blood vessel development’ (p-value = 2.61E−6, 17 genes), etc. (b) The enriched subordinate GO terms of ‘extracellular region part’ in the cellular components included ‘extracellular matrix’ (p-value = 8.74E−30, 46 genes), ‘proteinaceous extracellular matrix’ (p-value=3.44E-25, 39 genes), ‘collagen’ (p-value=1.79E-7, 8 genes). (c) This diagram shows the subordinate GO terms of ‘binding’ such as collagen binding, pattern binding and carbohydrate binding.

**Figure S3. A graph showing the correlation between the quantitative real-time PCR (QRT-PCR) data and the microarray data for 8 DEGs**

The full name of the genes are as follows; *thrombospondin 4 (THBS4), fibromodulin (FMOD), collagen*, *type XIV, alpha 1 (COL14A1)*, *cathepsin K (CTSK)*, *epidermal growth factor (EGF)-like repeats and discoidin I-like domains 3 (EDIL3)*, *lysyl oxidase (LOX,* *secreted frizzled-related protein 4 (SFRP4),* and *S100 calcium binding protein A4 (S100A4)*. R2 is 0.41.

**Figure S4. The MRI images of the remaining 8 CMT patients (#4~#11)**

We measured the mean color intensity of the SCM with and without CMT on the axial T1 weighted, pre-operational MRI image using the region of interest (ROI) method on the same axial T1 weighted image using the same ROI method. Red and green circles indicate the cross sectional areas of the SCM with and without CMT, respectively. The red arrows indicate the lowest signal areas of CMT.
